# Psychophysiological strategies for enhancing performance through imagery–skin conductance level analysis in guided vs. self-produced imagery

**DOI:** 10.1038/s41598-024-55743-w

**Published:** 2024-03-02

**Authors:** Dagmara Budnik-Przybylska, Paweł Syty, Maria Kaźmierczak, Jacek Przybylski, Łukasz Doliński, Marta Łabuda, Patryk Jasik, Adrian Kastrau, Selenia di Fronso, Maurizio Bertollo

**Affiliations:** 1 Division of Sport Psychology, Institute of Psychology, Faculty of Social Science, University of Gdańsk, Gdańsk, Poland; 2 Institute of Physics and Applied Computer Science, Faculty of Applied Physics and Mathematics, Gdańsk University of Technology, Gdańsk, Poland; 3 BioTechMed Center, Gdańsk University of Technology, Gdańsk, Poland; 4 Institute of Psychology, Faculty of Social Sciences, Division of Family Studies and Quality of Life, University of Gdańsk, Gdańsk, Poland; 5 Department of Biomechatronics, Faculty of Electrical and Control Engineering, Gdańsk University of Technology, Gdańsk, Poland; 6 Department of Medicine and Aging Sciences, Behavioral Imaging and Neural Dynamics Center, University “G. d’Annunzio” of Chieti-Pescara, Chieti, Italy

**Keywords:** Guided vs. self-produced imagery, Athletes, Skin conductance level, Clusterization, Dynamic time warping, k-Shape, Biophysics, Psychology

## Abstract

Athletes need to achieve their optimal level of arousal for peak performance. Visualization or mental rehearsal (i.e., Imagery) often helps to obtain an appropriate level of activation, which can be detected by monitoring Skin Conductance Level (SCL). However, different types of imagery could elicit different amount of physiological arousal. Therefore, this study aims: (1) to investigate differences in SCL associated with two instructional modalities of imagery (guided vs. self-produced) and six different scripts; (2) to check if SCL could differentiate respondents according to their sport expertise. Thirty participants, aged between 14 and 42 years (*M* = 22.93; *SD* = 5.24), with different sport levels took part in the study. Participants listened to each previously recorded script and then were asked to imagine the scene for a minute. During the task, SCL was monitored. We analysed the mean value, variance, slope and number of fluctuations per minute of the electrodermal signal. Unsupervised machine learning models were used for measuring the resemblance of the signal. The Wilcoxon signed-rank test was used for distinguishing guided and self-produced imagery, and The Mann–Whitney U test was used for distinguishing results of different level athletes. We discovered that among others, self-produced imagery generates lower SCL, higher variance, and a higher number of fluctuations compared to guided imagery. Moreover, we found similarities of the SCL signal among the groups of athletes (i.e. expertise level). From a practical point of view, our findings suggest that different imagery instructional modalities can be implemented for specific purposes of mental preparation.

## Introduction

Mental preparation is a very complex process because it involves adjusting to the challenging situations not only technically but also mentally^1^. Athletes need to be “in the zone” and achieve their optimal level of arousal for their optimal performance. From a psychophysiological perspective, they should be prepared to reach the right level of activation and self-regulate their behaviour before and during competitions^2^. Therefore, imagery is very often used because it not only improves motor skills and performance efficiency but also influences physiological arousal^3–5^. Although there is plenty of research linking imagery to sport success^6,7^, there is still a lack of research on how different instructions of imagery impact physiological arousal and self-regulation in a group of athletes with different levels of expertise. Both arousal and self-regulation have been linked to the skin conductance level (SCL)^8^. For this reason, in the current study, we investigated the relations between guided vs. self-produced imagery and SCL in a group of athletes.

Imagery is a multimodal cognitive simulation that helps to represent perceptual information in our minds without actual sensory input^5,9^. It is defined as “creation or re-creation of an experience generated from memorial information, involving quasi-sensorial, quasi-perceptual, and quasi-affective characteristics, that is under the volitional control of the imager, and which may occur in the absence of the real stimulus antecedents normally associated with the actual experience”^5^ (p. 19)”. From a psychophysiological perspective the bioinformational theory of Lang^10–12^ emphasises the role of stimuli (e.g., the imagined situation of starting in a competition induces certain physiological responses in individuals such as the modulation of the skin conductance level). A functional similarity between imagery and real execution (functional equivalence) was also revealed in other studies^13–18^. However, in previous models (e.g. RAMDIU^6^, PETTLEP^19^) authors stressed the importance of “how” to use imagery. Thus, not only the context but also the way of inducing imagined situations is important.

According to the literature novice or recreational athletes use less imagery and present a lower level of imagery ability than elite athletes^6,20^. Higher level athletes allocate a greater and higher commitment devoted to imagery practice compared to lower level athletes^21^. Moreover, research demonstrated that different perspective as well as modality should be differently used according to sport level. For example, internal and visual imagery focusing on trajectory of the ball and the target will be more effective for more advanced athletes who have huge knowledge about the service; adversely, focusing on the racket will be more effective for beginners^22^. Also, more advanced athletes present higher physiological responses in motor imagery compared to less advanced^23^.

In the literature at least two instructional modalities of imagery have been listed: guided and self-produced. Guided imagery is often used, among other fields, in medicine and rehabilitation to help patients in their recovery or control their emotional reactions^24–26^. It can be done through audio recordings, videos, or by means of the voice of counsellors to help the participant focus by using breathing techniques, progressive relaxation, as well as positive suggestions to relieve some somatic symptoms^27^. In guided imagery interventions, participants follow a script while imaging^28^. On the other hand, self-produced imagery scripts cause higher psychophysiological responses^29^, which may be linked to personal mental representation and the respondent’s understanding of the imagined scene^30^. The two instructional modalities (guided vs. self-produced) have been previously revealed to modulate brainwaves and specifically to induce higher high alpha and SMR in the case of guided imagery, whereas self-produced imagery might facilitate higher low alpha^31^. Self-produced imagery might facilitate higher relaxation; adversely, guided imagery may produce stronger attentional focus^25,32^. However, based on the existing literature there is a lack of information about what kind of instructional modality of imagery should be used for different levels of athletes. Therefore, there is a need to analyse this crucial issue.

Similarly to brainwaves, skin conductance level is another biomarker indicating the degree of mental or physiological arousal. This is one of the components of electrodermal activities (EDA), and it is an indicator of the activity of sweat glands innervated by the sympathetic branch of the autonomic nervous system^8^. The more the body is activated, the more sweat is secreted by these glands. The SCL is the EDA's overall tonic level, which relates to the slower-acting components and background characteristics of the signal (overall level, slow rise, slow dips over time). Consequently, it refers to a physiological response that is slow and may be graded. For this reason, changes in SCL are thought to reflect general changes in autonomic arousal. It is important to note that the slow nature of SCL changes generates a time-varying baseline in the signal. The dynamics of these changes may be different for different people as well as for several measurements of the same person.

The literature indicates that the EDA is related to the level of stress^33^ and can provide information on the emotional regulation ability in situations of stress coping^34,35^. Research on the relationship between the SCL and emotional states suggests that increased SCL is often interpreted as an indicator of emotional reactivity^36,37^. A high SCL may indicate severe difficulties in activating the parasympathetic nervous system to regulate the emotional state^35,38^. On the other hand, low SCL is interpreted as an indicator of fearlessness and/or emotional insensitivity^36^. Confirmation of the above statements seems to be provided by the results of a study, during which a reduction in skin conductance level and HR (heart rate) was observed after exposure to a stress factor in participants trained in the use of cognitive regulation^39^. Similar effects were obtained for participants after a single meditation session^40,41^.

Athletes must self-regulate their anxiety and arousal to adapt to new situations and/or different tasks. Accordingly, they can show similar emotional arousal during both optimal-and suboptimal-automatic performances (i.e., Type 1 and Type 4 performance states of the Multi Action Plan (MAP model); see Bertollo et al. for details)^42^. Pleasant feelings and similar SCLs characterise these states, but while the first is effortless and smooth, in the second, the performer is not very engaged and does not invest enough energy in task execution. The first ideal state can be easily undermined by different performance circumstances, and athletes can enter a suboptimal-controlled condition (i.e., Type 3) characterised by negative feelings, task-irrelevant focus, excessive reinvestment, and the highest SCL. However, self-regulation and attention to the core component of the action can lead to an optimal-controlled state (Type 2), typified by a slightly lower level of SCL compared to Type 3 performance and threat perceived as functionally optimal, although negative feelings.

Athletes differ in their degree of adaptation and self-regulation to new situations or different tasks. A number of studies relate to differences between elite and less advanced athletes, i.e. according to the neural and psychomotor efficiency^43–46^, in which experts have generally characterised an increased oscillation of the low alpha linked as a lower energy consumption. Moreover, experts develop an efficient organisation of task-related neural networks for specialised motor planning, but novices have problems filtering the information^47^. The study by Tremayne and Barry^48^ with shooters revealed that the experts’ skin conductance level and HR were lower than in novice shooters prior to the initiation of the shot. A similar pattern of HR and SCL decrease before short badminton serves was also observed by Fahimi and VaezMousavi^49^ in elite badminton players compared to beginners.

A question arises on whether two instructional modalities, such as, guided vs. self-produced imagery, may elicit different SCLs. Therefore, we have reanalysed the data collected in a previous study^3^ with different purposes and aimed to: 1) investigate differences in SCL associated with two instructional modalities of imagery (i.e., guided vs. self-produced) and six different scripts (e.g. training or competition environment); 2) check if SCL could differentiate respondents according to their sport expertise. Following these aims, we formulated three hypotheses. Self-produced imagery, as compared to guided imagery, is associated with a lower level of activation (i.e. SCL) (H1). Regardless of the SCL average it is important to analyse how individuals respond to stimuli and are able to adapt and self-regulate their behaviour to the demands of the environment. To this purpose, we analyse SCL slopes (calculated as the linear regression coefficient), hypothesising a negative slope in both instructional imagery modalities due to the process of habituation and familiarisation of the participants with the task (H2a). We additionally analyse the SCL variability and the number of fluctuations in the signal. Thus, it is expected that self-produced imagery is associated with a higher level of SCL variance and number of fluctuations, i.e., adaptation/self-regulation as compared to guided imagery (H2b). Moreover, we check if all the previous SCL indices as well as the pattern of the SCL signal (similarities between the signals of the participants) can differentiate respondents according to their level of expertise (H3).

## Results

### Data pre-processing

Raw SCL signal has been cleaned according to the 1.5 IQR (interquartile range) rule: points outside the range [Q1 − 1.5*(Q3 − Q1), Q3 + 1.5*(Q3 − Q1)], where Q1 and Q3 are the first and third quartiles, respectively, have been removed. Then, normalisation of the SCL signal has been performed with respect to the mean of the 5 s period before each script, so that the normalised SCL signal (called nSCL) represents the percentage SCL change during the script. This approach has been used before in several studies^3,52^. The test scene involving squats was not included in the primary analysis but was utilised to assess the statistical power of the study. The standardised mean difference, calculated by comparing the mean nSCL in the test scene with that in the six scenes examined (0.023), along with the standard deviation (0.03), yielded a standardised mean difference of 0.77. At a significance level of 5% (0.05), this results in a statistical power of 91.35%.

### Data processing and analyses

#### SCL indices in self-produced vs guided imagery according to the level of expertise of the athletes

The mean value (nSCL_mean), variance (nSCL_var), slope (nSCL_slope) and number of fluctuations per minute (nSCL_nfsc) of the nSCL signal have been calculated for each person and scenario, divided into guided and self-produced parts. The slope of the signal has been calculated using linear regression. All of the above statistics were also tested for the outliers (using the 1.5 IQR method), and the outliers have been removed. Following the Storm et al.^53^, fluctuation in the SCL signal can be defined as a peak with a minimum amplitude of 0.02 μS and a slope rate < 2 μS. In our study, the nSCL_nfsc value is defined as the number of skin conductance fluctuations during the 1 min. period. Basic statistics (means and standard deviations) of the resulting datasets are collected in Table 1. None of the resulting datasets have a normal distribution or homogeneity of variance. This consequently led to the selection of non-parametric Wilcoxon tests of potential differences between these two groups for repeated measurements. In other words, the tests allow us to verify the null hypothesis, that these two groups are equal.

**Table 1:** Basic statistics (means and standard deviations) of the mean value (nSCL_mean), variance (nSCL_var), slope (nSCL_slope) and number of fluctuations per minute (nSCL_nfsc) of the nSCL signal, separately for guided and self-produced parts of the scenarios.

	Guided imagery		Self-produced imagery	
	*M*	*SD*	*M*	*SD*
nSCL_mean	− 0.85	2.20	− 3.05	4.92
nSCL_var	2.09	2.11	2.54	2.02
nSCL_slope	− 0.047	0.072	− 0.049	0.053
nSCL_nfsc	1.36	1.08	3.0	1.94

*N* = 180 in each case (30 persons × 6 scenarios).

In Table 2, analogous basic statistics have been collected for additional division of the participants into two groups, according to their sport caliber (Tiers 1–3 and Tier 4), as well as separately for guided and self-produced parts of the scenarios. To test for differences in these groups (by rejecting the null hypothesis that groups are equal), again, non-parametric tests (Mann–Whitney U tests, since this time we deal with independent samples) have been selected due to the limited normality and homogeneity of the resulting datasets.

**Table 2:** Basic statistics (means and standard deviations) of the mean value (nSCL_mean), variance (nSCL_var) and slope (nSCL_slope) of the nSCL signal, separately for guided and self-produced parts of the scenarios and sport level of the participants.

	Guided imagery				Self-produced imagery			
	Tiers 1–3		Tier 4		Tiers 1–3		Tier 4	
	*M*	*SD*	*M*	*SD*	*M*	*SD*	*M*	*SD*
nSCL_mean	− 0.64	2.69	− 1.57	1.36	− 2.54	5.61	− 3.77	3.06
nSCL_var	2.82	3.21	1.31	1.10	2.87	2.22	1.45	0.88
nSCL_slope	− 0.024	0.112	− 0.068	0.046	− 0.044	0.062	− 0.054	0.038
nSCL_nfsc	1.50	1.01	0.90	0.74	3.32	1.99	2.27	1.58

*N* = 126 for Tiers 1–3, *N* = 54 for Tier 4.

SCL indices in self-produced vs. guided imagery results of the Wilcoxon signed-rank test for distinguishing guided and self-produced imagery for selected statistics of the nSCL signal are collected in Table 3. We can notice (with statistical significance), with connection to Table 1, that:

- self-produced imagery generates lower SCL compared to guided imagery,
- the variance (the measure of variability) of the SCL signal is higher in self-produced imagery,
- the number of fluctuations (peaks) in the SCL signal is also higher in self-produced imagery.

**Table 3:** The Wilcoxon signed-rank test for distinguishing guided and self-produced imagery.

	*W*	*p*
nSCL_mean	3037.0	< 0.001*
nSCL_var	4596.0	0.045*
nSCL_slope	5011.0	0.677
nSCL_nfsc	1944.0	< 0.001*

*Indicates p < 0.05.

The same conclusion can be derived from the direct counting of the participants with higher (or lower) values of investigated variables in the majority of scenarios. Particularly, for nSCL_mean, we can see that 24 participants have a lower average SCL in self-produced imagery than in guided imagery, 4 have the opposite, and 2 have almost the same value. The higher variance (nSCL_var) in the self-produced imagery has 17 participants, 8 the opposite, and 5 without noticeable change. Finally, 28 participants have a higher number of fluctuations in the self-produced imagery (only 2 have the opposite).

For the slope of the SCL (nSCL_slope), the participants are not significantly outnumbered in any part of the scenario, which indirectly confirms the lack of statistical significance in the Wilcoxon test. Anyway, we can observe that slopes were negative in general, and also (insignificantly) lower in the self-produced part.

In Table 4, results of the Mann–Whitney U test for distinguishing Tiers 1–3 from Tier 4, separately for guided and self-produced imagery, are collected. Analysing the results, we can see that in guided imagery, all of the signal features can distinguish the participants with respect to Tiers 1–3 and Tier 4, with statistical significance. In the self-produced imagery, we can say that only about the variance and number of fluctuations in the SCL. Looking back at Table 2, we can observe, that:

1. in guided imagery:
2. mean SCL level is higher for athletes from Tiers 1–3, than from Tier 4,
3. variability of the SCL signal is higher in Tiers 1–3,
4. slope of the SCL signal is higher in Tier 4,
5. the number of peaks in SCL is again higher in Tiers 1–3;
6. in self-produced imagery:
7. variability of the SCL signal is higher in Tiers 1–3,
8. the number of peaks in SCL is again higher in Tiers 1–3,

**Table 4:** The Mann–Whitney U test results for distinguishing Tiers 1–3 from Tier 4, separately for guided and self-produced imagery.

	Guided imagery		Self-produced imagery	
	*U*	*p*	*U*	*p*
nSCL_mean	3090.0	0.025*	3020.0	0.176
nSCL_var	3226.5	0.004*	3421.5	< 0.001*
nSCL_slope	3404.0	0.004*	2999.0	0.457
nSCL_nfsc	4251.0	< 0.001*	4503.5	0.001*

*Indicates p < 0.05.

and, without statistical significance (but the values in tiers are clearly different):

- mean SCL level is higher for athletes in Tiers 1–3,
- the slope of the SCL signal is steeper in Tier 4.

As presented above, the results are consistent in both guided and self-produced imagery.

#### Similarities of the SCL signal in self-produced and guided imagery according to the athletes' level of expertise

To check if—beside the basic statistics—also the shape of the nSCL signal could differentiate the investigated groups defined before (guided vs. self-produced, also with respect to the sport level), we have applied two algorithms which allowed us to find similarities between SCL time series.

Dynamic Time Warping (DTW) is an algorithm used to measure the similarity between two sequences of time signals of different lengths^54^. The method is unique in such a a way that it takes into account that the positions of maxima in two time series may differ slightly from each other, so that the series is warped to match each other in a nonlinear way. It can be based solely on shape or, additionally, on absolute signal differences. This, as opposed to simply comparing signals, allows a more accurate determination of similarity, due to the consideration of individual differences in physiological response. In our study, the method, based on the shape only, has been used to calculate pairwise similarity between nSCL signals, within each part of scenarios (guided and self-produced) on the numerical scale (0–100, where 0 means no similarity, and 100 means perfect match). These scores, by averaging over all scenarios, are used for the calculation of the similarity between particular persons. This, in turn, is used to calculate the average similarity between participants within the Tiers 1–3, and Tier 4, to verify if the SCL shape could differentiate these Tiers.

Another approach to that problem relies on the k-Shape clusterization. This iterative machine learning method is analogous to well–known k-means clustering, but applied to time series^55^. Clustering is done into two clusters (expecting that they will correspond to investigated Tiers), for all participants and for each scenario, separately for guided and self-produced parts. The quality of clusterization into two clusters has been measured by the Silhouette score^56^. That score takes values from -1 to 1, and positive values, preferably higher than 0.5, prove correct clusterization. Next, results from all scenarios were joined by using consensus clustering^57^. In this procedure, it has been counted how many times a particular pair of participants have been assigned to the same cluster (0–12). That, in turn, allowed us to find a global division into clusters, taking into account all scenarios.

The next step of our analyses were focused on finding similarity of the SCL signal itself, among athletes presenting a similar level of expertise in their sport discipline. These analyses were conducted without any a priori assumptions regarding the division of participants, so they can be classified as the unsupervised machine learning.

**Figure 1:**
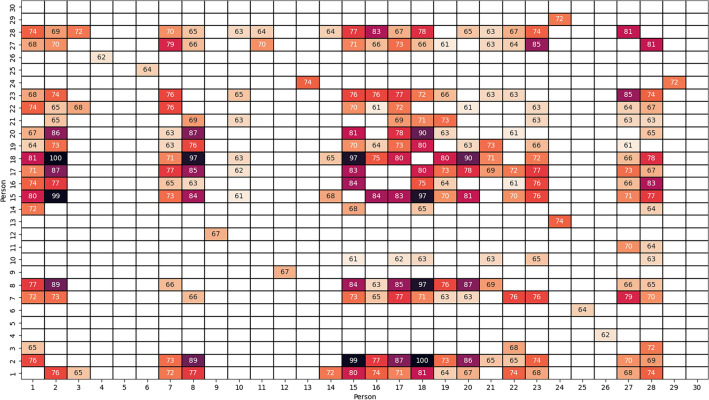
Average pairwise similarity between persons in guided imagery, calculated using DTW method, in 0–100 scale. Higher values means greater similarity of the SCL signals for particular persons. Only values higher than 60 are shown.

**Figure 2:**
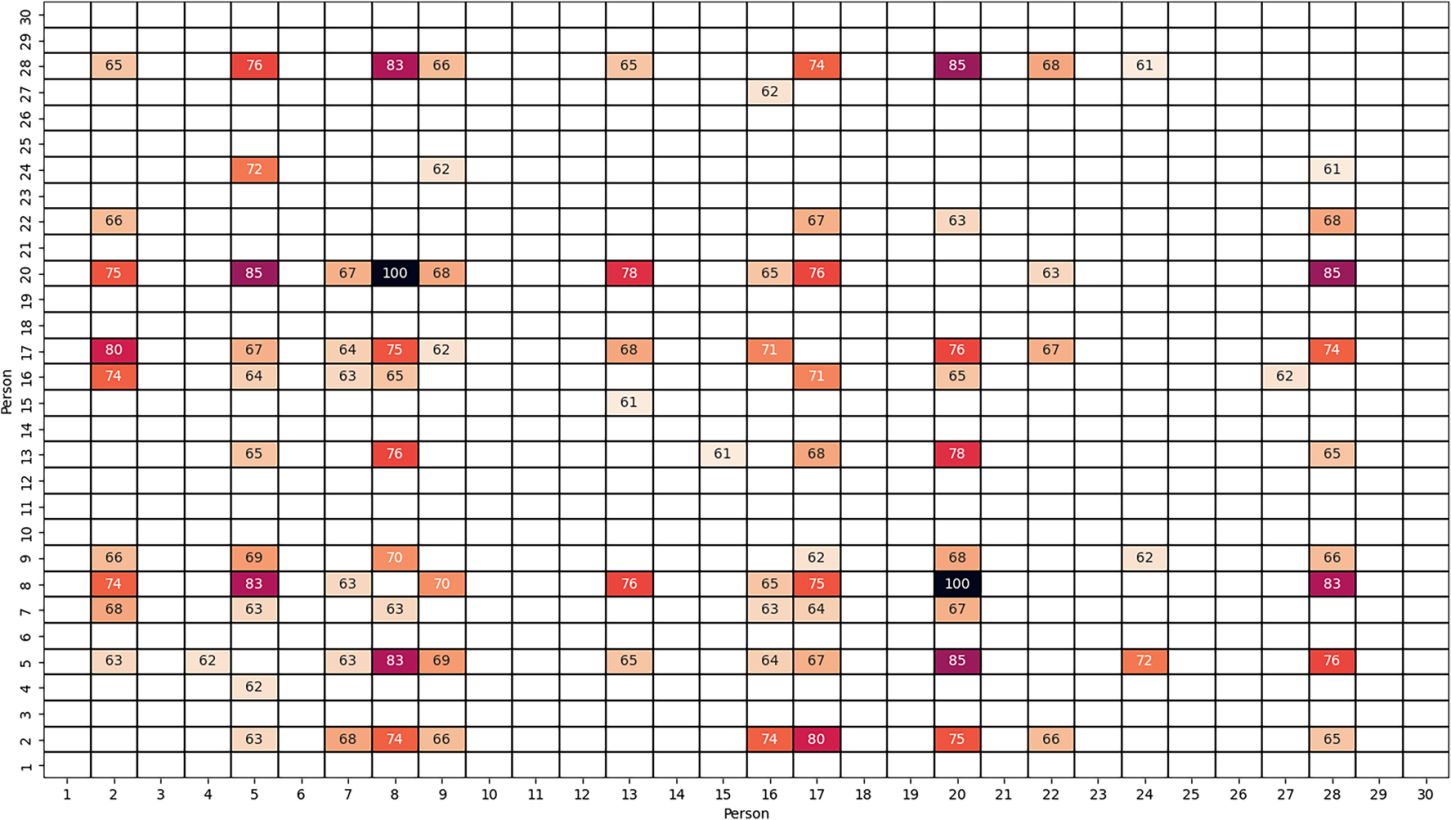
Average pairwise similarity between persons in self-produced imagery, calculated using DTW method, in 0–100 scale. Higher values means greater similarity of the SCL signals for particular persons. Only values higher than 60 are shown.

DTW pairwise similarity has been calculated for each scenario, separately for guided and self-produced imagery, in the (arbitrary) scale from 0 to 100. The obtained results, averaged over considered scenarios, are collected in Figs. 1 and 2.

Additionally, average similarity between persons belonging to Tiers 1–3 and 4 has been calculated. Considering the guided imagery, average similarity between participants is found to be 39.69% for Tiers 1–3, and 47.00% for Tier 4. In self-produced imagery, these values are 32.10% and 54.11%, respectively. The obtained results indicate that SCL responses of athletes representing international sport level (Tier 4) are more similar to each other, than responses of athletes from Tier 1–3. This is true in both guided and self-produced imagery.

**Figure 3:**
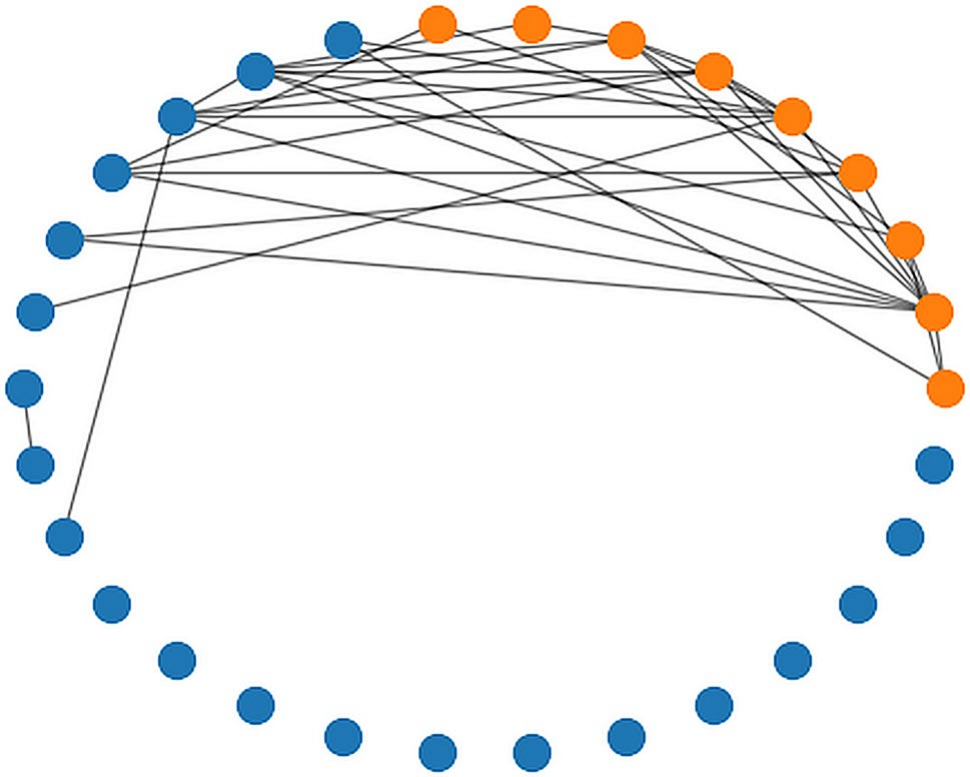
Graph of connections between participants, obtained using the k-Shape clustering method. It was assumed that participants are related (connected by the line) if they were assigned to the same cluster at least 10 out of 12 times. Blue points—participants from Tiers 1–3; orange points—participants from Tier 4.

Results of the k-Shape clusterization into two clusters, applied to all participants, separately for each scenario and type of imagery, are collected in Table 5. For example, in the guided imagery, Training session scenario, a set of participants 1, 2, 3, 5, 8, 9… was classified to cluster 1, and persons 4, 6, 7, 10… to cluster 0, respectively. Silhouette score for that case (0.44) shows that the quality of clusterization is quite good. Average Silhouette score is 0.52, which indicates that separation between clusters is acceptable. In the next step, it was counted how many times (0–12) a particular pair of persons have been assigned to the same cluster (so-called consensus clustering). This is visualised at the connectivity graph in Fig. 3, for participants who were assigned to the same cluster at least 10 out of 12 times. It is clearly seen that most of the connections are between the orange points, representing Tier 4. There are only 6 blue points, representing Tiers 1–3, connected to the other orange points, and 3 other blue points connected to each other. The rest of the blue points are not connected at all. This can be interpreted in the way that the shape of the SCL signal can distinguish the group of athletes of the highest sport achievements (Tier 4), from the others.

**Table 5:** k-Shape clustering results. Assignments of particular persons (1–30) to clusters (1, 0) for each scenario, separately for guided and self-produced imagery, together with the Silhouette scores.

	Scenario	Persons (1–30) assignments to clusters (1, 0)	Silhouette score
Guided	Training session	1 1 1 0 1 0 0 1 1 0 1 1 0 1 1 1 0 1 1 1 0 1 1 0 0 0 0 1 0 1	0.44
	Your home venue	1 1 1 0 0 1 1 1 1 1 0 1 1 1 1 1 1 1 0 1 0 1 1 1 0 0 1 1 1 0	0.50
	Successful competition	0 1 1 0 1 0 1 1 0 1 0 0 1 0 1 0 1 1 1 1 1 0 1 1 1 0 1 1 1 1	0.43
	Fitness activity	1 1 0 1 1 1 1 1 0 1 1 1 0 1 1 1 1 1 1 1 1 1 1 0 1 0 1 1 0 1	0.63
	Slow start	1 1 1 0 0 0 1 1 0 0 1 1 1 1 1 1 1 1 1 1 1 1 0 1 0 0 1 1 1 1	0.58
	Start in a high level championship	1 1 1 0 1 1 1 1 0 1 1 0 0 1 1 1 1 1 1 0 1 0 1 0 1 0 1 1 0 1	0.60
Self-produced	Training session	0 0 0 0 0 1 1 0 0 1 1 0 0 0 0 0 0 1 0 0 0 1 1 0 1 1 0 0 0 1	0.50
	Your home venue	0 0 1 0 0 0 0 0 0 0 1 0 0 0 0 0 0 1 0 0 1 0 1 0 0 1 0 0 0 0	0.52
	Successful competition	1 1 1 0 0 0 1 0 0 1 0 0 0 0 0 1 0 0 0 0 0 0 1 0 0 1 1 0 1 1	0.46
	Fitness activity	0 0 1 0 0 1 0 0 0 0 1 0 1 1 1 0 0 0 1 0 1 0 1 0 0 0 0 0 0 0	0.53
	Slow start	1 1 1 1 1 1 1 1 1 0 1 0 1 1 1 1 1 0 0 1 1 1 0 0 1 1 0 1 1 1	0.56
	Start in a high level championship	0 1 0 0 1 1 1 1 0 1 1 0 1 0 1 1 1 1 1 1 1 1 0 0 0 0 1 1 1 1	0.51
Average Silhouette score			0.52

Average Silhouette score is 0.52.

The above procedure has been repeated for clusterization into three and four clusters, giving lower average Silhouette scores (0.36 and 0.26, respectively). It shows that two is the optimal number of clusters when it comes to k-shape clustering.

## Discussion

The main aim of our study was to investigate the association between SCL and two instructional modalities of imagery (guided vs. self-produced). We confirmed our first hypothesis associated with this goal and verified that a person's arousal, as measured by SCL, is lower during self-produced imagery than during guided imagery. However, as expected, we observed negative slopes in both situations as a consequence of the adaptation and familiarisation with the task (H2a). Additionally, SCL variance and number of fluctuations were higher in self-produced than guided imagery, which confirmed the H2b hypothesis. Moreover, we found that expertise level has an effect on SCL signals with the more expert athletes (Tier 4) being faster to self-regulate their psychophysiological state (steeper slope of SCL) and more able to cope with the demands of the stimuli (less variability in SCL) with no differences in the two imagery modalities.

The results related to the H1 hypothesis, are in line with the bioinformational theory of Lang^10–12^ and the previous findings in which the cortical activity of the athletes, when performing guided or self-produced imagery, was assessed using EEG^31^. In that case, we found that guided imagery induced higher high alpha and SMR (usually associated with selective attention), whereas self-produced imagery facilitated higher low alpha (associated with global resting state and relaxation). Similarly, in the current study athletes produced lower values of SCL during self-produced imagery and therefore seemed to be more relaxed and less activated. Such instructional modality may help to keep more control over the image and therefore be less stimulating to the athletes. However, it was also revealed that body response to self-produced imagery can be linked to personal indices like general imagery, sport anxiety or neuroticism^3^. Our findings are also in line with the very recent study by Hu and Yu^58^ about visual imagery in which respondents assessed the subjective experience of imagery and vividness weaker in self-generated imagery than cue-induced (triggered by external cues). Our result is partly in contrast to the study of Wilson et al.^29^ in which self-produced imagery caused higher psychophysiological responses. However, in that study participants were asked to generate simple movements and only EMG (electromyography) was monitored, but our scripts were more complex and could indeed generate different emotions. Therefore, our findings significantly contribute to the development of “how” imagery should be used from simple to complex situations.

Drawing on the MAP model perspective^42^, self-produced imagery should be implemented to train more automatic responses with lower arousal and lower activation of the nervous system, as in a flow-like performance state (i.e., Type 1 performance) also linked to lower energy consumption and positive feelings. On the other hand, when athletes need to control their performance and consequently use a higher level of effort and activation, they need to train and/or prepare themselves using guided imagery.

The lower SCL average in self-produced imagery could also be due to the fact that respondents were disengaged. However, it should be emphasised that each person completed the same task under both instructional modalities in seven different scripts; therefore, we are able to examine intrapersonal comparisons. We confirmed that in guided imagery respondents typically presented higher SCL which meant that they were more engaged and followed the instruction. This is in line with studies in medicine where guided imagery is used to empower patients to actively participate in their recovery or control their emotions^25^.

According to the H2a hypothesis, which referred to adaptation and self-regulation in imagery, negative slopes were seen in both situations. This may be explained by the fact that the majority of respondents were accustomed to the new situation and there was a kind of adaptation/habituation to the stimuli. With great caution, participants became calmer regardless of instructional modality. Another explanation could be that most respondents were not able to maintain the focus on the imagined situation all the time. The question arises for the future practical application about the duration of the imagery session in the imagery training^59^ and conditions determining the duration of the imagined picture^60^. Since our study includes very complex tasks and high freedom in imagery, therefore we could analyse higher spectrum of the mental imagery process.

According to the Multi-States (MuSt) theory for emotion and action-regulation^2^ individual appraisal has a key role in self-regulation. Therefore, athletes should use self-regulation strategies to achieve in-zone conditions. Referring to the H2b hypothesis, the variance and the number of fluctuations were higher in self-produced imagery. It is likely that participants actively adjusted to the imagined situation. Similarly to the HRV (heart rate variability), which is treated as the indicator of self-regulatory capacity^61,62^, the higher the level of SCL variance and the higher the number of fluctuations, the more work the body needs in order to adjust to the situation.

The third aim was to check whether SCL signals could differentiate respondents according to the sport level. We also confirmed our third hypothesis H3. More advanced athletes obtained a lower level of SCL average in both instructional modalities compared to less advanced. We could say that they were less aroused compared to lower level athletes. Moreover, advanced athletes obtained lower levels of SCL variability and lower number of peaks in SCL in both instructional modalities of imagery. Therefore we could say that they need effort to adjust to what they imagined. Additionally, the slope of the SCL signal was higher in more advanced athletes which meant that they became familiar with the new situation faster. The results were consistent in both guided and self-produced imagery. These findings are in line with neural and psychomotor efficiency theory^44,63^ and previous studies^48,49^.

Another innovative method which was used for verifying H3 was unsupervised and supervised machine learning models which revealed that more advanced athletes presented a similar pattern in SCL as the whole signal than less advanced in both instructional modalities. This is a unique finding which confirms that higher level athletes seem to be similar to each other in SCL during imagery. Indeed, there is plenty of research concerning differences between elite and novice athletes in imagery ability^64,65^, use^66^ or motor imagery^67,68^. However, this is the first study using an objective innovative method in which similarity in SCL signals were revealed during imaging the expanded mental imagery scripts.

### Limitations and strengths of the study

A limitation of our study was undoubtedly the rather small and inhomogeneous sample, therefore, our study should be treated as exploratory in nature. We are aware that the results could be differentiated depending on the imagery modality and perspective used but also task characteristics^6,18,20,69–73^ and individual’s imagery ability can depend on the imagery modality^6,18,20,69^. However, each participant imagined seven situations, which required complex and time-consuming procedure. All situations were selected from previously existing and validated questionnaires. Additionally, the inclusion criteria was to take part in sport competitions regardless of their level. The strength of this study is the length of the scripts, because previous studies often used very short and simple scripts.

This is a first and unique study using different instructional imagery modalities during which skin conductance level was monitored and analysed by innovative methods such as unsupervised machine learning models. We used DTW for measuring the similarity between two sequences of time signals of different lengths^54^ but also k-Shape clusterization, as an iterative, machine learning method applied to time series^55^. Thanks to those mentioned methods we found that athletes characterised by a similar level of expertise present a similar pattern of SCL level.

To our knowledge, this is the first study where the complex SCL indices were analysed during the different instructional modalities of imagined scenes. We also incorporate new methods of analysis to reveal similarities in SCL signals according to athletes’ sport caliber. Moreover, it is an extension of the knowledge on “how” imagery should be used from simple to complex situations.

From an applied point of view, our study could be used in the field for choosing the best mental training strategies. For instance, guided instructional modality should be implemented in situations which demand focus, mental effort and higher activation. Adversely, self-produced imagery should be used in situations which are less challenging to help athletes to regulate their emotions. Such instructional modality can be treated as a kind of “first aid kit”. According to the concept of systematic desensitisation (as an exposure therapy)^74^, used in stressful situations, which involves gradually exposing the individual to the anxiety-provoking stimuli in a controlled and relaxed environment, self-produced imagery, as the intervention, may be used for similar purposes. This is also in line with the Yerkes-Dodson law^75^ when athletes should learn how to adjust their arousal to the situation and obtain the medium level which finally is the most effective. To conclude, to activate the athlete during imagery in more complex situations it is advised to use guided imagery, adversely, if we want to regulate one's arousal state it is advisable to use self-produced imagery.

## Methods

### Participants

Thirty participants took part in our study (14 females and 16 males), aged between 14 and 42 years with an average age (M) of 22.93 years (*SD* = 5.24) (below 20 years: *N* = 5, 20–29 years: *N* = 22, 30 and more years:* N* = 3). The majority of the participants (*N* = 25) were emerging adults according to the definition by Willoughby et al.^50^ typically lasting between the ages of 18 and 29. Their sports experience varied, ranging from 2 to 20 years, with a mean (*M*) of 10.15 years (*SD* = 4.75) (5 and less years: *N* = 7, 6–10 years: *N* = 6, more than 10 years: *N* = 17) and diverse backgrounds in terms of sport disciplines (combat sports (*N* = 11), individual sports (*N* = 7), team sports (*N* = 12) and sport levels: according to McKay et al.^51^ Tiers 1–3 *N* = 21 (which represent Recreationally Active, Trained/Developmental and Highly Trained/National Level) and Tier 4 (International Level). All participants filled in two imagery questionnaires and all obtained at least 19 points out of 30 in general imagery ability (general tendency to use imagery) *M* = 24.57, *SD* = 3.06, and 1905 out of 4430 in Total SIAM (*M* = 3042.5, *SD* = 473.81). None of the participants obtained the lowest level in both questionnaires, so we decided to leave the whole group for further analysis.

Written informed consent was obtained from the athletes to participate in the study, ensuring the protection of their personal data. For participants below the age of majority, informed consent was obtained from a parent and/or legal guardian. The investigation followed the ethical principles regarding human experiments as defined in the Declaration of Helsinki and received approval from the local Institutional Review Board (University of Gdańsk, 11/2015).

### Procedure

Participants were presented with a pre-recorded script (guided imagery) and were then asked to imagine the scene (self-produced imagery) they listened to for one minute. We incorporated seven sport-related scenarios including one test scene: imagining performing 10 squats and six situations used from two existing questionnaires on imagery in sport: the Sport Imagery Ability Measure (SIAM^76^) questionnaire in the Polish adaptation^77^ and the Imagination in Sport Questionnaire (ISQ)^64^. Throughout this task, we continuously monitored participants’ physiological responses using a multimodal device. Six scenes (Fitness Activity, Successful Competition, Your Home Venue, Training Session, Slow Start, Start in a High Level Championship (precompetitive routine)) with a target imagery were presented randomly, and brief pauses were inserted between each block to activate the participants. The whole procedure lasted approximately fifteen minutes. Following the imagery session, participants completed a set of questionnaires—inter alia ISQ, SIAM. During the task, several biofeedback parameters, including SCL were monitored using the Biofeedback Expert 2000 System equipment by Schuhfried.

Sport Imagery Ability Measure (SIAM^76^) in the Polish adaptation^77^, which is a 72-item self-report questionnaire that uses six sport-related scenes to examine the dimensions of vividness, control, duration, ease, and speed of generation: the visual, auditory, olfactory, gustatory, tactile, and kinesthetic senses; and the experience of emotion. Participants are given 60 s to image each scene. They are then required to respond to 12 items designed to assess imagery dimensions. Responses are made responses ranging from no feeling to very clear feeling. The relevant sensory item scores for the six scenes (e.g. six vividness scores, six visual scores) are added together to calculate the twelve subscale scores and the total score.

The Imagination in Sport Questionnaire (ISQ^64^) is a multidimensional 51-item measurement tool that consists of seven subscales, i.e., physiological feelings, modalities, ease/control, perspective, affirmations, visual, and general. The participants imagined a competitive situation for 60 s in as detailed and realistic a manner as possible. Then the participants respond to the 51 items and rate the different aspects of the image on a scale from 1 (not at all) to 5 (completely so). All subscales (except one that was named “general”) were related to the imagined situation: i.e., situational imagery. The “general” subscale consisted of six questions and was developed separately to assess the general tendency to use imagery: i.e., general imagery.

### Example of imagery scripts

Successful Competition. Imagine you are competing in a specific event or match for your sport. Imagine that you are at the very end of the competition and the result is going to be close. You pull out a sensational move, shot, or effort to win the competition. Take notice of what you can see around you, the sounds you hear, and the feel of any muscles moving. Do you get the sensation of any smells or tastes? Can you feel the equipment and surfaces you are using? Do you get an emotional feeling from this activity? Now you have 60 s to create and experience your image of the scene.

### Data analysis

In the present study, we focused on the SCL only. The RAW data, collected at a frequency of 40 Hz and expressed in units of microsiemens (μS) were cleansed using standard techniques and normalised^78^. Then, the mean value (nSCL_mean), variance (nSCL_var), slope (nSCL_slope) and number of fluctuations per minute (nSCL_nfsc) of the nSCL signal have been calculated for each person and scenario, divided into guided and self-produced parts. The slope of the signal has been calculated using linear regression. Analysis has been done using proprietary Python code with some external libraries (including dtaidistance^79^ and tslearn^80^).

## Data Availability

The dataset analysed during the current study is available in the Bridge of Knowledge Open Data Repository, 10.34808/7pfg-1w13.
